# The value of chest magnetic resonance imaging compared to chest radiographs with and without additional lung ultrasound in children with complicated pneumonia

**DOI:** 10.1371/journal.pone.0230252

**Published:** 2020-03-19

**Authors:** Philip Konietzke, Jan Mueller, Felix Wuennemann, Willi L. Wagner, Jens-Peter Schenk, Abdulsattar Alrajab, Hans-Ulrich Kauczor, Mirjam Stahl, Marcus A. Mall, Mark O. Wielpütz, Olaf Sommerburg

**Affiliations:** 1 Department of Diagnostic and Interventional Radiology, University Hospital of Heidelberg, Heidelberg, Germany; 2 Translational Lung Research Center Heidelberg (TLRC), German Center for Lung Research (DZL), Heidelberg, Germany; 3 Department of Diagnostic and Interventional Radiology with Nuclear Medicine, Thoraxklinik at University of Heidelberg, Heidelberg, Germany; 4 Department of Diagnostic and Interventional Radiology, Section Pediatric Radiology, University Hospital of Heidelberg, Heidelberg, Germany; 5 Department of Translational Pulmonology and Division of Pediatric Pulmonology & Allergy and Cystic Fibrosis Center, University Hospital of Heidelberg, Heidelberg, Germany; 6 Department of Pediatric Pulmonology, Immunology and Intensive Care Medicine, Charité-Universitätsmedizin Berlin, Berlin, Germany; 7 Berlin Institute of Health (BIH), Berlin, Germany; Northwestern University Feinberg School of Medicine, UNITED STATES

## Abstract

**Introduction:**

In children with pneumonia, chest x-ray (CXR) is typically the first imaging modality used for diagnostic work-up. Repeated CXR or computed tomography (CT) are often necessary if complications such as abscesses or empyema arise, thus increasing radiation exposure. The aim of this retrospective study was to evaluate the potential of radiation-free chest magnetic resonance imaging (MRI) to detect complications at baseline and follow-up, compared to CXR with and without additional lung ultrasound (LUS).

**Methods:**

Paired MRI and CXR scans were retrospectively reviewed by two blinded readers for presence and severity of pulmonary abscess, consolidation, bronchial wall thickening, mucus plugging and pleural effusion/empyema using a chest MRI scoring system. The scores for MRI and CXR were compared at baseline and follow-up. Furthermore, the MRI scores at baseline with and without contrast media were evaluated.

**Results:**

33 pediatric patients (6.3±4.6 years), who had 33 paired MRI and CXR scans at baseline and 12 at follow-up were included. MRI detected significantly more lung abscess formations with a prevalence of 72.7% compared to 27.3% by CXR at baseline (p = 0.001), whereas CXR+LUS was nearly as good as MRI. MRI also showed a higher sensitivity in detecting empyema (p = 0.003). At follow-up, MRI also showed a slightly better sensitivity regarding residual abscesses. The overall severity of disease was rated higher on MRI. Contrast material did not improve detection of abscesses or empyema by MRI.

**Conclusion:**

CXR and LUS seem to be sufficient in most cases. In cases where LUS cannot be realized or the combination of CXR+LUS might be not sufficient, MRI, as a radiation free modality, should be preferred to CT. Furthermore, the admission of contrast media is not mandatory in this context.

## Introduction

Pneumonia is an important cause of morbidity and mortality in children worldwide [1]. There is a reported annual incidence of 150.7 million children worldwide, 7–13% of which need hospital admission [2]. Although around 95% of cases of pneumonia-related deaths occur in developing countries, there is also a considerable incidence of severe pneumonia in high-income countries with a rising number of children hospitalized due to complicated lung infection [2–4]. Though antibiotics are sufficient in 80–90% of patients with pulmonary abscesses, there are still some patients in whom conservative treatment fails, making early drainage or surgery a life-saving intervention [5–7]. According to current guidelines, chest x-ray (CXR) is typically the first imaging modality employed in hospitalized children when treatment does not improve the clinical condition and/or a parapneumonic effusion is suspected [8]. CXR beyond the initial procedure on admission is barely necessary. Follow-up is only necessary if cough persists and/or other signs suggest that the child has not completely recovered [9, 10]. Current evidence also supports lung ultrasound (LUS) as an imaging alternative for the diagnosis of childhood pneumonia showing a sensitivity of 96% and a specificity of 93% [11]. Computed tomography (CT) is not implemented in routine work-up for children with complicated pneumonia due to its relatively high radiation exposure, but expert consensus suggests the use of CT to determine the extent of parenchymal disease, abscess formation and to guide surgical procedures [8, 12, 13]. During a prolonged course of complicated pneumonia serial CXR or CT may be required, which further increases radiation exposure [12]. A radiation-free alternative to CT for cross-sectional imaging is magnetic resonance imaging (MRI). MRI showed to be superior to CXR in detecting pulmonary abscess formation/necrosis in children with complicated pneumonia and was also successfully implemented in the workup of pediatric lung disease such as cystic fibrosis [14–18]. There is also extensive data from the application of MRI in cystic fibrosis, and in this context infiltrates or consolidations found on MRI have been compared against paired CT examinations, revealing a high concordance of 100% in children and adults [19]. Furthermore, pulmonary MRI has been proven to be feasible and comparable to CT in adults, by detecting infiltrates in febrile neutropenic patients and performing similar to CT in the detection of pulmonary findings suggestive for pneumonia in immunocompromised patients [20, 21]. Literature on the use of the CXR in pediatric pneumonia is broadly available, whereas the data on the use of the MRI is limited [22]. The aim of this study was to evaluate the potential of MRI in detecting complications and typical morphological findings in complicated pneumonia and to evaluate the role of MRI in therapy monitoring.

## Materials and methods

### Patient recruitment

This retrospective study was approved by the local ethics committee and informed consent for data processing was waived.

The patients were treated according to current guidelines on community acquired pneumonia [23, 24]. The decision on diagnostic imaging, the duration of intravenous or total antibiotic treatment as well as on intervention was made by the treating pediatrician. The diagnosis of severe pneumonia was based on clinical signs (hypoxia, tachypnoae, grunting, chest indrawing and/or crackels on auscultation) as well as on blood testing (C-reactive protein (CrP) >5 mg/l, white blood cell count (WBC) >4-10/ml). Detection of the causing agent was always attempted from blood, airway secretion, or drainage fluid before an empiric antibiotic therapy was started. In most cases patients were treated initially with oral aminopenicilline and/or after admission in the hospital with a second- or third-generation cephalosporine. In cases of unclear clinical course of pneumonia and/or suspected atypical bacteria, a combined therapy was started by adding a macrolide. Clindamycin was provided in addition to β-lactam antibiotic therapy when clinical, laboratory, or imaging characteristics were consistent with infection caused by *Staphylococcus aureus*. The management in case of pleural effusion and/or empyema followed current guidelines [8]. Insertion of chest drain was considered if substantial parapneumonic effusion could be shown and pneumonia treatment failed at 48 hours. Intrapleural fibrinolytics were recommended if parapneumonic effusion turned into thick fluid with septations or empyema. Surgery was considered when chest tube drainage failed and/or the symptomatic child developed an organized empyema. Follow-up MRI was indicated if cough, fever, CrP, and WBC persisted and follow-up CRX did not show any improvement.

The inclusion criteria for the present study were (1) diagnosis of complicated pneumonia, (2) age <18, (3) complete lung MRI protocol (S1 Table), (4) CXR within ±5 days of the initial MRI scan, and (5) if follow-up MRI was available, CXR within ±5 days of the follow-up MRI scan.

### MRI and CXR imaging

MRI (1.5T Magnetom Avanto, Siemens Healthineers AG, Erlangen, Germany) was performed in supine position. The protocol usually starts with a balanced steady-state free precession (bSSFP) sequence, acquired in free-breathing to provide an overview of respiratory movements. The further protocol is adapted depending on patient size and ability to breath-hold, containing a T1-weighted gradient-echo (VIBE) or turbo spin-echo (TSE) as well as a T2-weighted half-fourier single-shot turbo spin-echo (HASTE) or a motion-insensitive multi-shot turbo spin-echo (BLADE) sequences. In children unable to breath-hold, a T1-weighted fast spin echo (FSE) sequence and averaging may be used (S1 Table). The intravenous application of gadolinium-based contrast media was done by a power injector as recommended [25]. The common side effects of intravenous contrast injection and dose as well as national prescription regulations were considered with respect to patient age. Routine sedation is usually necessary in preschool children (<6 years). The preferred medication at our institution is rectally or orally administered chloral hydrate under monitoring by paediatrician [14]. The overall room time for the imaging protocol was approximately 15 minutes. All examinations were visually inspected for absence of significant motion artefacts and inclusion of all parts of the chest by a senior chest radiologist. CXR (Philips Bucky Diagnost CS; Philips; Amsterdam; Netherlands) was acquired in standing/sitting (n = 27) or supine position (n = 6). Lateral views were not made. The protocol was adjusted to the age of the patient. The following protocols were used (1) 5 years (79kV, 1.25mAs, 11.2ms), (2) 10 years (96kV, 1.60mAs, 17.0ms) and (3) 15 years (125kV, 1.00mAs, 13.8ms).

### Semiquantitative image analysis

Two radiologists with 5 years of experience in thoracic imaging analyzed the images using a picture archiving and communication software (PACS) workstation (Centricity, Version 4.0; General Electrics, New York, USA). Images were provided in a randomized fashion to the readers. All MRI and CXR exams were retrospectively reviewed for the presence of pulmonary abscess, consolidation, bronchial wall thickening, mucus plugging and pleural effusion/empyema using a modified previously evaluated chest MRI scoring system [14, 26–28]. The following modifications were introduced: 1) Combined scoring for pulmonary necrosis and abscess formation because the first is a known risk factor for developing cavitary lung lesions [29–31] and 2) Combined scoring of pleural effusion and empyema. All findings were rated for each lobe in MRI (right and left upper and lower lobe, middle lobe and lingula) or lung fields in CXR (right and left upper, middle and lower lung) to enable allow comparability between MRI and CXR. The percent size of the area affected was scored: score = 0 (no abnormality), score = 1 (<50% of the lobe/lung-field involved), or score = 2 (≥50% of the lobe/lung-field involved). In MRI, pleural effusion/empyema was rated as score = 1 if effusion size was smaller than 1 cm and score = 2 if effusion size was larger than 1 cm. The sum of the subscores resulted in a global score for both imaging modalities. Retrospective scoring of lung ultrasound (LUS) examinations was not possible due to non-standardized examination protocols. Due to this, only the final diagnosis from the medical report was used for the present study.

### Statistical analysis

Statistical analysis was performed using SPSS 22 Statistics (IBM, Armonk, New York, USA). Simple weighted kappa coefficients were used to assess intra- and interreader agreement. Kappa was interpreted as an indication that agreement was slight when 0 to 0.2, fair when greater than 0.2 to 0.4, moderate when greater than 0.4 to 0.6, substantial when greater than 0.6 to 0.8, and near perfect when greater than 0.8 to 1.0, in accordance with Landis and Koch [32]. Consensus was achieved by calculating the mean values of scores assigned by the two readers. The means ± standard deviation for all subscores were calculated separately for MRI and CXR as well as all imaging features. Prevalence and subscores were compared with Wilcoxon signed rank test due to non-parametric distribution. A p-value of <0.05 was considered statistically significant. An additional analyses using the Bonferroni-Holm method for multiple testing was performed, which did not change the number of significant results.

## Results

### Patient cohort

**Fig 1:**
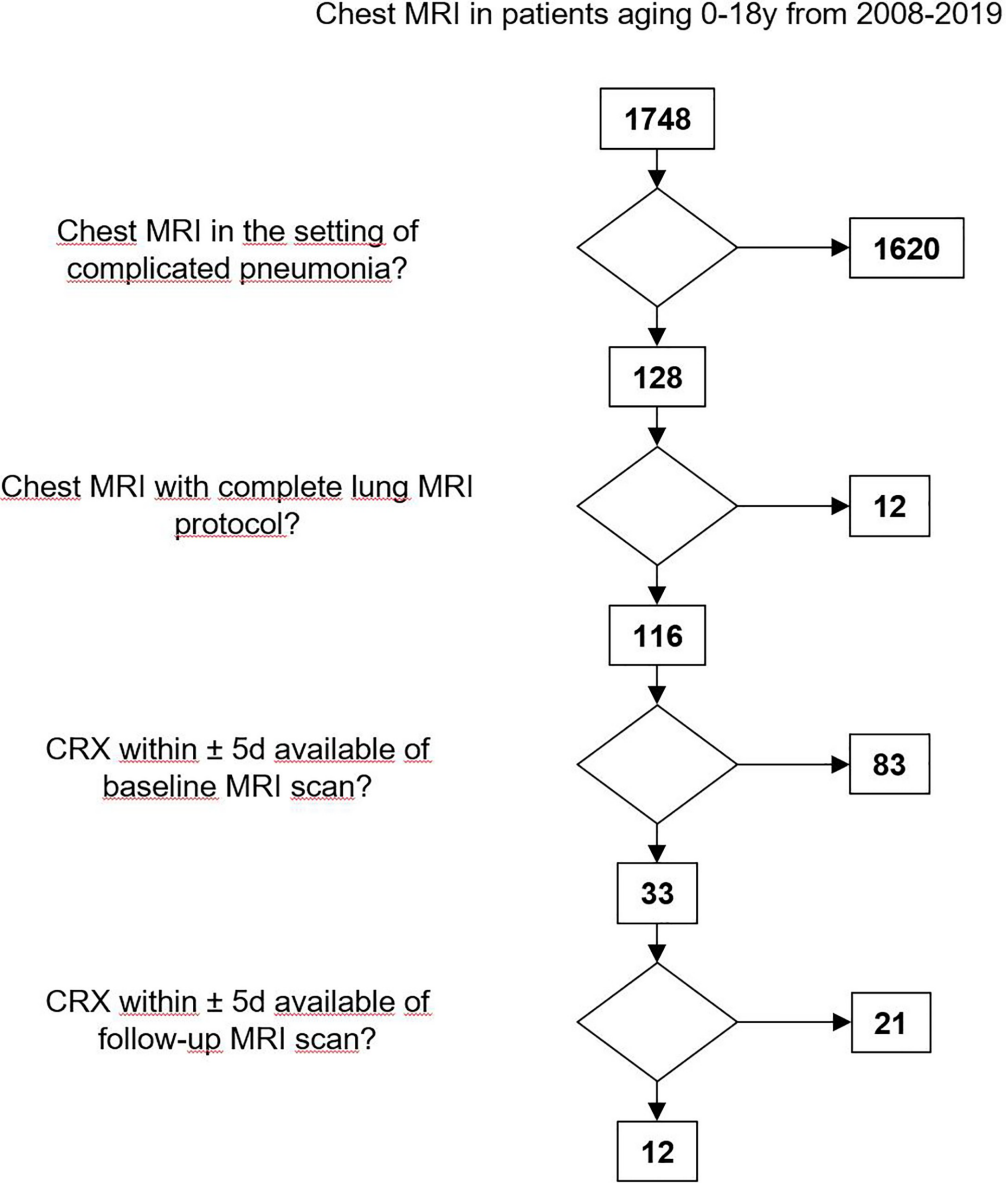
Flowchart for patient recruitment. A database research encompassing the years 2008–2019 identified 1748 patients who underwent chest MRI. 128 patients had chest MRI in the setting of complicated pneumonia. Out of these, 12 patient were excluded due to incomplete lung MRI protocol or bad image quality. 33 patients had CXR within ±5 days of the baseline MRI scan and 12 patients had also CXR within ±5 days of the follow-up MRI scan.

A database research encompassing the years 2008–2019 identified 1748 patients who underwent chest MRI. Out of these 128 patients had chest MRI in the setting of complicated pneumonia. In total, 33 patients aged 6.3±4.6 years could be recruited for this study (Table 1). CXR was performed in standing/sitting (n = 33) and supine position (n = 10) within 1.45±1.42 days of MRI acquisition. In 30 patients gadolinium-based contrast agent was applied intravenously, while 3 patients received no contrast agent due to impaired kidney function (GFR <25 ml/min). Additionally, lung ultrasound (LUS) was performed in 27 patients within 1.11±2.15 days of MRI acquisition. In 12 patients follow-up MRI was performed 18.17±15.05 days after baseline MRI. The time interval between MRI and CXR was 2.01±1.58 days at follow-up. These patients were imaged after adjustment of antibiotic therapy (n = 3), renewed chest drain (n = 1) or to exclude complications in persistent clinical symptoms (n = 8). (Fig 1).

**Table 1:** Patient characteristics.

Subjects	
N	33
Age (years)	6.3 ± 4.6
Male/Female	17/16
Weight (kg)	20.5 ± 12.5
Height (cm)	111.0 ± 26.8
BMI (kg/m^2^)	15.6 ± 2.2
Streptococcus pneumoniae detected (%)	9
Staphylococcus aureus detected (%)	2
Other bacteria detected (%)	4
Treated with drainage or surgery (%)	18

Baseline patient characteristics given as median and standard deviation. BMI = body mass index.

### Intra- and interreader agreement

The best intra-reader agreement was achieved for consolidation and for pleural effusion/empyema with κ = 0.89 and κ = 0.91 for MRI and κ = 0.94 and κ = 0.85 for CXR. Bronchial wall thickening showed substantial agreements for MRI with κ = 0.64 and only fair agreement for CXR with κ = 0.49. The lowest agreement was found for mucus plugging with κ = 0.64 for MRI, whereas mucus plugging was not found in CXR. Reader 1 achieved overall slightly higher levels of intra-reader agreement than reader 2 for nearly all subscores. According to this, the agreement for the global score was also slightly higher for Reader 1 with κ = 0.80 for MRI and κ = 0.92 for CXR, compared to κ = 0.89 and κ = 0.72 for Reader 2. The levels of agreement where overall slightly better in CXR than for MRI (Table 2).

**Table 2:** Intra-reader agreement for MRI and CXR scores.

Intrareader agreement	MRI			CXR		
**Reader 1**	**Read 1**	**Read 2**	**κ**	**Read 1**	**Read 2**	**κ**
Abscess/necrosis	1.70±1.40	1.45±1.46	0.85	0.33±0.68	0.39±0.81	0.88
Consolidation	4.82±2.52	4.67±2.46	0.91	3.48±1.96	3.30±1.98	0.96
Bronchial wall thickening	2.03±1.38	1.76±1.39	0.76	0.10±0.29	0.05±0.17	0.49
Mucus plugging	0.39±0.69	0.21±0.54	0.53	0	0	-
Pleural effusion/empyema	4.30±2.33	4.18±2.32	0.94	3.30±2.29	3.00±2.22	0.89
Global score	13.24±4.93	12.27±5.00	0.80	7.21±3.70	6.73±3.72	0.92
**Reader 2**						
Abscess/necrosis	1.67±1.36	1.42±1.46	0.83	0.42±0.82	0.36±0.73	0.72
Consolidation	4.91±2.49	4.76±2.47	0.87	3.45±1.91	3.27±1.93	0.92
Bronchial wall thickening	2.39±1.50	1.73±1.38	0.52	0.09±0.29	0.03±0.17	0.49
Mucus plugging	0.55±0.86	0.27±0.57	0.40	0	0	-
Pleural effusion/empyema	4.45±2.24	4.15±2.26	0.88	3.27±2.23	2.94±2.20	0.89
Global score	14.00±4.93	11.97±5.08	0.72	7.24±3.77	6.61±3.70	0.89

Means ± standard deviation for all findings in complicated lower airway tract infection in n = 33 pediatric patients are shown for magnetic resonance imaging (MRI) and for chest x-ray (CXR). Simple weighted kappa coefficients (κ) were calculated for intrareader agreement for both Reads and Reader.

The best interreader agreement for MRI and CXR was achieved for consolidation with κ = 0.97 and κ = 0.98, and for pleural effusion/empyema with κ = 0.97 and κ = 0.95. Abscess/necrosis was also near perfect with κ = 0.97 for MRI and κ = 0.86 for CXR. Bronchial wall thickening showed a higher agreement for MRI with κ = 0.83 and only substantial agreement for CXR with κ = 0.66. The agreement on the global score was also near perfect with κ = 0.92 for MRI and κ = 0.96 for CXR (Table 3).

**Table 3:** Inter-reader agreement for MRI and CXR scores.

Interreader agreement	MRI			CXR		
	Reader1	Reader2	κ	Reader1	Reader2	κ
Abscess/necrosis	1.58±1.38	1.56±1.38	0.97	0.36±0.73	0.39±0.75	0.86
Consolidation	4.74±2.43	4.83±2.43	0.97	3.39±1.95	3.36±1.90	0.98
Bronchial wall thickening	1.89±1.32	2.06±1.26	0.83	0.06±0.20	0.07±0.20	0.66
Mucus plugging	0.30±0.58	0.38±0.65	0.87	0	0	-
Pleural effusion/empyema	4.24±2.31	4.30±2.23	0.97	3.15±2.22	3.11±2.18	0.95
Global score	12.76±4.85	12.98±4.85	0.92	6.97±3.67	6.92±3.69	0.96

Means ± standard deviation for all findings in complicated lower airway tract infection in n = 33 pediatric patients are shown for magnetic resonance imaging (MRI) and chest x-ray (CXR). Interreader agreement was calculated with simple weighted kappa coefficients (κ) between Reader 1 and Reader 2.

### Prevalence and severity of imaging findings at baseline

Looking at the prevalence of imaging findings, MRI revealed significantly more pulmonary abscess/necrosis formations with 72.7% compared to CXR with 27.3% (p = 0.001). The prevalence of consolidation and pleural effusion/empyema was not significantly different (p = 1.000 and p = 0.125). The prevalence of bronchial wall thickening was 97% in MRI compared to 27.3% in CXR (p = 0.001), whereas mucus plugging was found in 66.7% of the patients in MRI but was not detected in CXR (p = 0.001).

**Fig 2:**
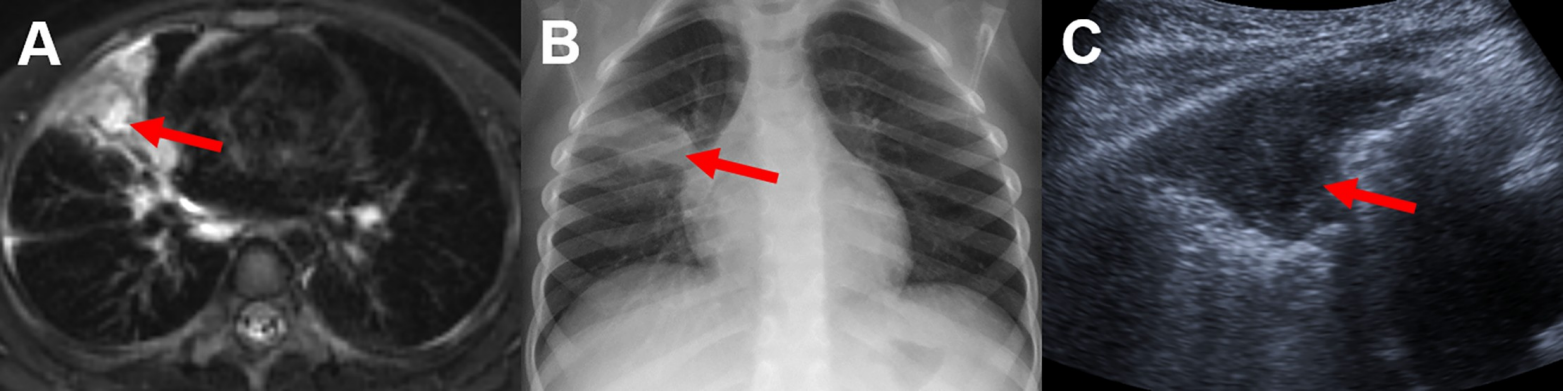
Contrast-enhanced MRI, CXR and LUS study of a 3-year old child. T2 weighted MRI (A) demonstrates an abscess formation within the consolidation in the middle lobe (red arrow), not detected by CXR (B) showing only consolidation (red arrow). In LUS (C) the abscess demarcates barley and was not diagnosed initially (red arrow).

Compared to the sole use of CXR, additional LUS increased the prevalence of abscess/necrosis to 55.6% (p = 0.109), which was still lower than the 77.8% with MRI. The prevalence of pleural effusion/empyema increased to 88.9%, which was comparable to MRI (p = 0.895). If pleural effusion and empyema were differentiated, the prevalence of empyema was significantly higher in MRI compared to the sole use of CXR, with 74.1% compared to 11.1% (p = 0.003). The additional use of LUS increased the prevalence of empyema from 11.8% to 63.1%, which was nearly as good as for MRI (Fig 2 and Table 4).

**Table 4:** Prevalence of morphologic findings in MRI and CXR at baseline.

Morphologic findings	MRI	CXR	p	MRI	CXR+ LUS	p
Abscess	72.7	27.3	0.001	77.8	55.6	0.109
Consolidation	100	97	1.000	100	96.3	1.000
Bronchial wall thickening	97	27.3	0.001	96.3	0	0.001
Mucus plugging	36.4	0	0.001	33	0	0.004
Pleural effusion/empyema	93.9	81.8	0.125	92.6	88.9	0.895

Prevalence in percent (%) for morphologic findings in complicated lower airway tract infection comparing magnetic resonance imaging (MRI) with chest x-ray (CXR) in n = 33 patients and MRI with chest x-ray CXR + lung ultra sound (LUS) in n = 27 patients.

**Fig 3:**
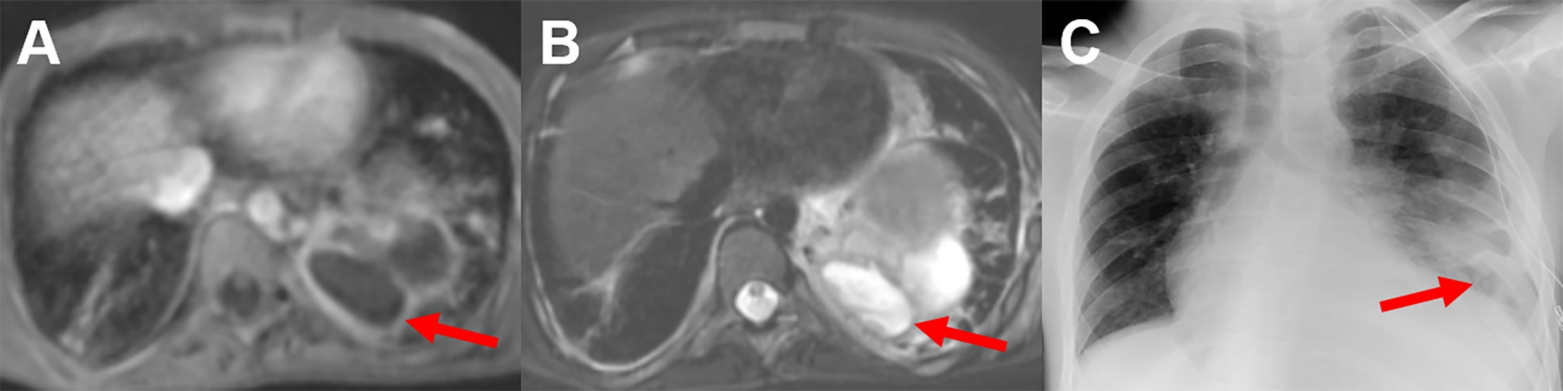
Contrast-enhanced T1 weighted sequence in comparison with T2 weighted sequence of a 7-year-old child. Both MRI sequences clearly delineate an abscess formation in the left lower lobe. On T1-weighting imaging (A) the abscess is characterized by the missing central contrast uptake and peripheral rim enhancement (red arrow). On T2-weighting (b), the necrotic center of the abscess has a high signal intensity, whereas the thick capsule shows a lower intensity (red arrow). The corresponding CXR (c) shows a consolidation in the left lower lung field as well as the abscess (red arrow). The example shows that the extension of abscess formations might be underestimated in CXR.

The severity of the morphologic findings was rated higher in MRI, which is represented by significant higher subscores. According to this, the global score was also significantly higher in MRI compared to CXR, with 12.95±4.83 and 6.95±3.68, respectively (p<0.001) (Fig 3 and Table 5).

**Table 5:** Scores for morphologic findings in MRI and CXR at baseline.

Morphologic findings	MRI	CXR	p
Abscess/necrosis	1.57±1.38	0.38±0.73	0.001
Consolidation	4.79±2.42	3.38±1.92	0.001
Bronchial wall thickening	1.98±1.26	0.06±0.20	0.001
Mucus plugging	0.34±0.61	0	0.001
Pleural effusion/empyema	4.27±2.26	3.13±2.20	0.001
Global Score	12.95±4.83	6.95±3.68	0.001

Subscores for morphologic findings in magnetic resonance imaging (MRI) and chest x-ray (CXR) in lower airway tract infection and global score in n = 33 pediatric patients with complicated pneumonia. Subcores were compared with Wilcoxon signed rank test due to non-parametric distribution. A p-value of <0.05 was considered statistically significant.

### Prevalence and severity of image findings at follow-up

**Fig 4:**
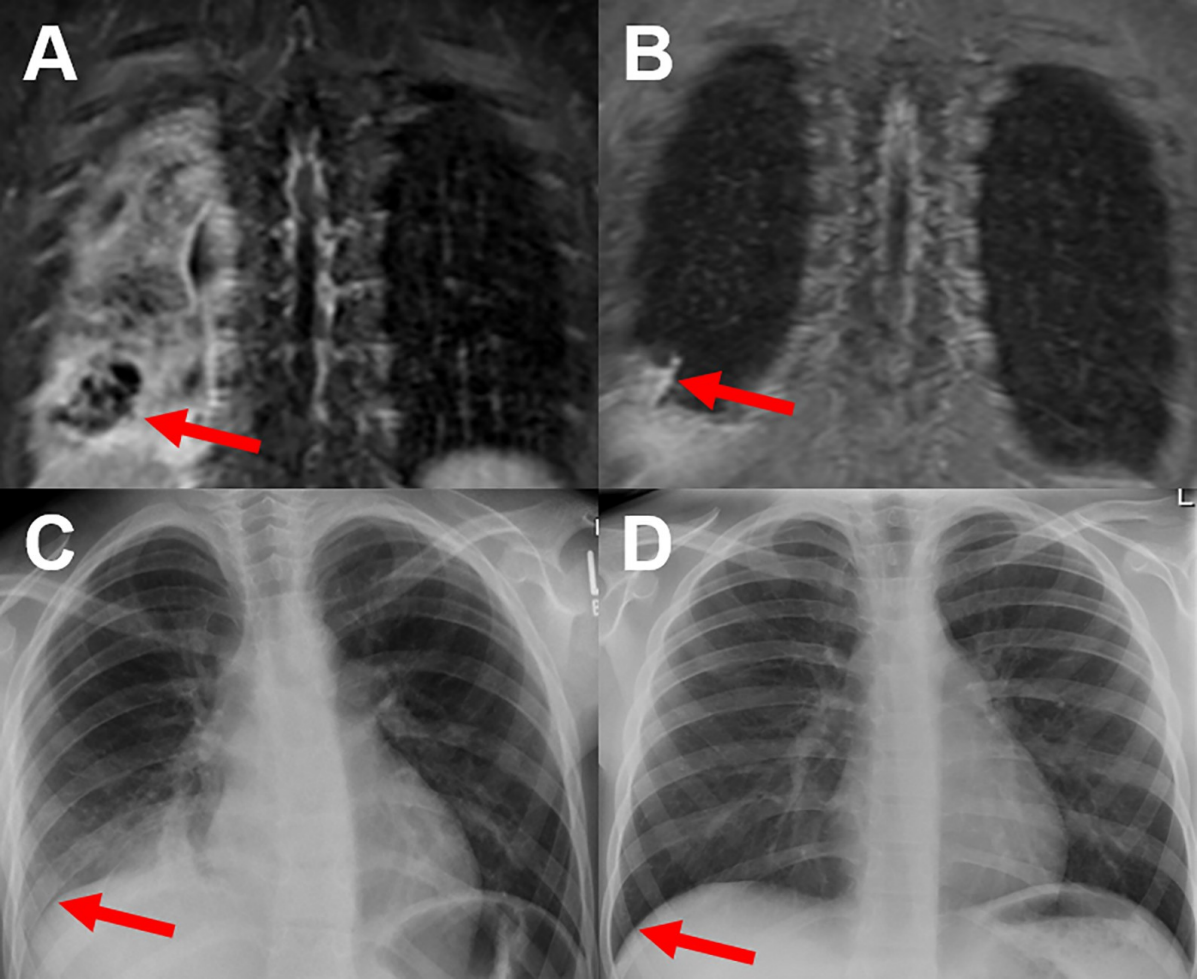
Comparison of T2-weighted sequences and CXR of a 9-year-old child at baseline and follow-up. MRI shows an abscess formation in the right lower lobe at baseline (red arrow) (A), which has regressed under therapy at follow-up (red arrow) (B). The corresponding CXR (C) shows a slight reduction in transparency in the right lower lung field without any clear evidence of an abscess (red arrow). At follow-up, CXR (D) shows no pathological findings not delineating the full extent of residual inflammatory changes (red arrow).

At follow-up exams, the prevalence of abscess/necrosis decreased from 83.3% to 66.7%, whereas it did not change in CXR (Fig 4). The prevalence for consolidations and pleural effusion/empyema did not change for MRI and decreased by -25% in CXR (p = 0.250). The prevalence of bronchial wall thickening and mucus plugging tended to decrease on MRI, whereas bronchial wall thickening increased slightly for CXR (p = 0.500). There was no mucus plugging in CXR (Table 6).

**Table 6:** Prevalence of findings in MRI and CXR at follow-up.

Morphologic findings	MRI			CXR		
	CT1	CT2	p	CT1	CT2	p
Abscess/necrosis	83.3	66.7	0.500	33.3	33.3	-
Consolidation	100	100	-	100	75	0.250
Bronchial wall thickening	100	91.7	0.941	8.3	25	0.500
Mucus plugging	41.7	33.3	0.813	0	0	-
Pleural effusion/empyema	100	100	-	100	75	0.250

Prevalence in percent (%) for morphologic findings in complicated lower airway tract infection comparing magnetic resonance imaging (MRI) with chest x-ray (CXR) in n = 12 patients at follow-up.

**Fig 5:**
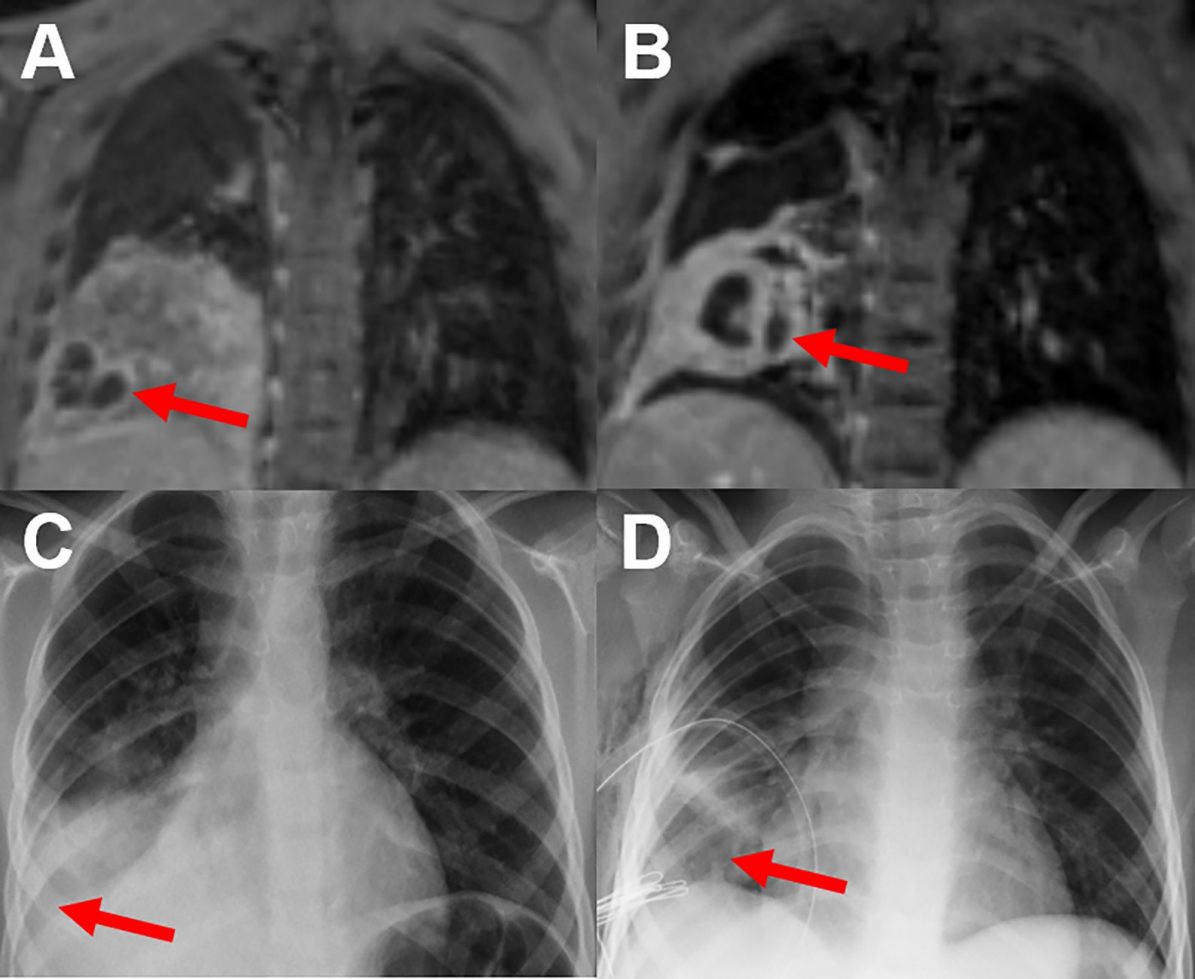
Comparison of T2-weighted sequences and CXR of an 11-year-old child at baseline and follow-up. MRI shows an abscess formation in the right lower lobe at baseline (red arrow) (A). The surrounding inflammatory changes regressed under therapy, whereas the abscess formation increased at follow-up (red arrow) (B). The abscess formation was not detected by CXR (red arrows) (C+D). Furthermore, the extension of pathologic findings was underestimated by CXR (D).

The subscores for abscess/necrosis decreased non-significantly by 0.54 (p = 0.275) for MRI and by 0.25 for CXR (p = 0.313). The subscores for consolidation and pleural effusion/empyema showed a significant decrease for MRI (p = 0.005; p = 0.019), whereas the subscores for CXR decreased only significantly for consolidation (p = 0.016). The subscores for bronchial wall thickening (p = 0.240) and mucus plugging (p = 0.469) tended to decrease also for MRI at follow-up, whereas it tended to increase for bronchial wall thickening for CXR (p = 0.250). The global score decreased significantly for MRI and CXR at follow-up (p = 0.012, p = 0.016) (Fig 5 and Table 7).

**Table 7:** Differences in scores for morphologic findings in MRI and CXR between baseline and follow-up.

Morphologic findings	MRI			CXR		
	Baseline	Follow-up	p	Baseline	Follow-up	p
Abscess/necrosis	1.96±1.27	1.42±1.56	0.275	0.52±0.82	0.27±0.43	0.313
Consolidation	4.83±1.92	2.81±2.19	0.005	3.48±1.01	1.77±1.73	0.016
Bronchial wall thickening	3.08±1.16	2.33±1.88	0.240	0.04±0.14	0.25±0.47	0.250
Mucus plugging	0.60±0.89	0.35±0.60	0.469	0	0	-
Pleural effusion/empyema	4.94±1.70	3.10±1.90	0.019	3.85±1.92	2.23±1.85	0.105
Global Score	15.42±4.65	10.02±6.41	0.012	7.90±2.67	4.52±3.58	0.016

Subscores at follow-up for morphologic findings in magnetic resonance imaging (MRI) and chest x-ray (CXR) in lower airway tract infection and global score in n = 12 pediatric patients with complicated pneumonia. Subcores were compared with Wilcoxon signed rank test due to non-parametric distribution. A p-value of <0.05 was considered statistically significant.

### Value of intravenous contrast media

**Fig 6:**
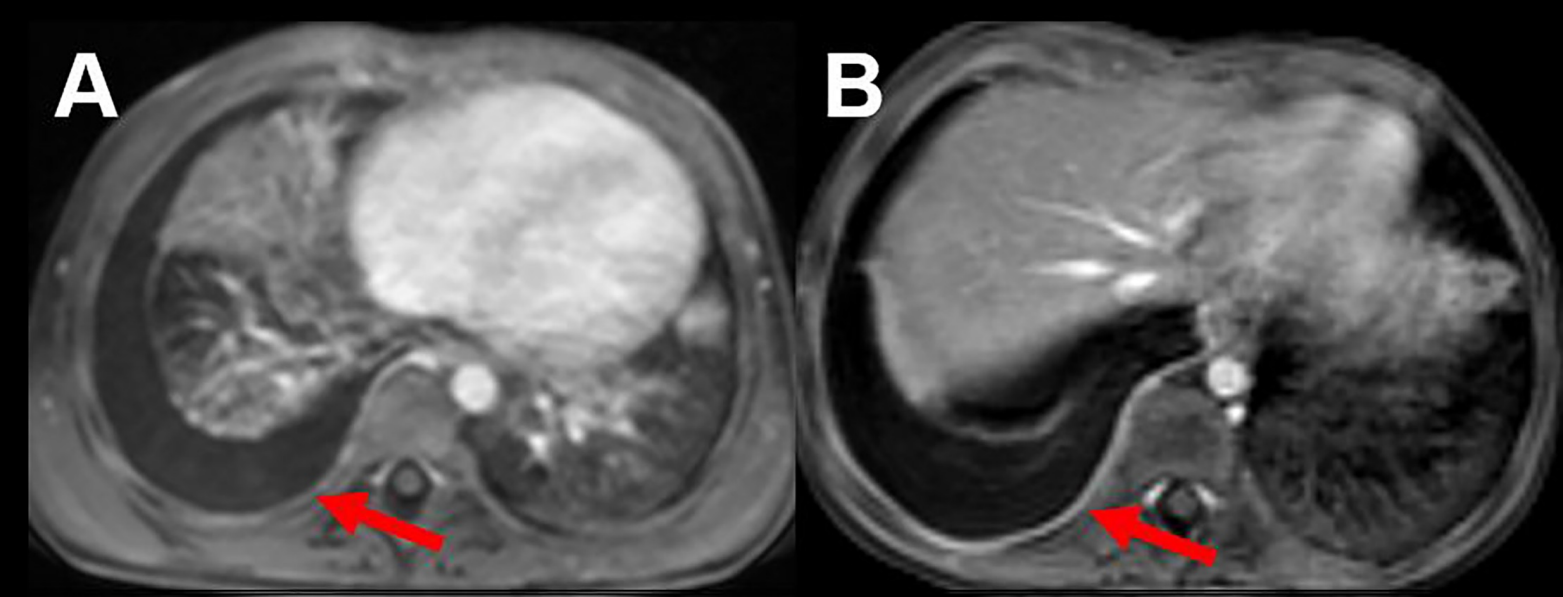
Contrast-enhanced T1 weighted sequence in in a 7-year-old child with pleural effusion and a 6-year-old child with pleuritis. Pleural effusion on the right side without pathologic contrast enhancement of the pleura (red arrow) (A). In comparison, also right sided pleural effusion, but with pathologic enhancement of the pleura, suggestive for an inflammatory process (red arrow) (B).

In 30 patients gadolinium-based contrast agent was applied intravenously. The number of abscess formations/necrosis detected by MRI was not influenced by the application of intravenous contrast as the additional review of a T1-weighted contrast-enhanced sequence did not alter the number of abscess formations/necrosis noted. In most cases the administration of contrast agents had no added value regrading the differentiation of pleural effusion and empyema since, all empyems showed septations in T2w-weighted sequences. Only one patient had pleural enhancement without septations in the pleural effusion (Fig 6).

## Discussion

Pneumonia is considered the leading cause of death in children under the age of five years worldwide. A gold standard for its diagnosis is currently missing, despite several clinical guidelines are available [1, 33]. Chest radiograph (CXR) is a widely employed test and typically the first imaging modality employed in hospitalized children. Literature on the use of the CXR in pediatric pneumonia is broadly available, whereas the data on the use of the MRI is limited [22]. The aim of this study was to evaluate the potential of MRI in detecting complications in severe pneumonia, as well as its role in therapy monitoring.

A modified chest MRI scoring system, which was used for scientific purposes in previews studies was used to compare MRI and CXR+(LUS) [14, 26–28]. The scoring system included the following morphological findings (1) pulmonary abscess/necrosis, (2) consolidation, (3) bronchial wall thickening, (4) mucus plugging and (5) pleural effusion/empyema. In the date analysis prevalence, severity of findings as well as intra- and interreader agreement was evaluated.

Pulmonary abscesses/necrosis manifest in CXR as cavities that may occur within areas of consolidation or might be isolated. In our study MRI revealed significantly more pulmonary abscess formations at baseline than CXR (p = 0.001). This indicates that CXR can reveal larger cavities and abscesses, but minor changes might avoid visual detection. These findings are in accordance with prior studies and provide further evidence that MRI is superior to CXR in detecting pulmonary abscess and necrosis formation [8, 18]. The severity of the morphologic findings was rated higher in MRI at baseline and follow-up, showing that the extent of disease might be underestimated in CXR. The intra- and interreader agreement was also slightly better in MRI, indicating a higher diagnostic certainty. If supplementary LUS was used the prevalence of abscess/necrosis increased to 55.6% (p = 0.109). Nonetheless, the prevalence of abscess/necrosis was still higher in MRI, although the difference was not significant anymore. The reason for this might be, that to identify pneumonia by LUS, a consolidation needs to reach the pleura and needs to be detected within an intercostal space [34]. Therefore, the combination of CXR and LUS has a high sensitivity for peripheral abscesses within consolidations, whereas the central parts of the lung and abscesses without large consolidations might be difficult to assess. We also observed that while administration of intravenous contrast agent improves the assessment of vitality/perfusion of lung tissue and allows a better evaluation of possible pleural lesions, it does not affect the sensitivity for pulmonary abscess formation/necrosis in MRI. The abscess wall typically appears hypointense compared to the fluid-isointense center of the abscess formation and can be clearly identified on T2-weighted images. In the clinical context the diagnosis of lung abscess formation is important, since it seems to be associated with a higher admission rate to the intensive care unit, while also bearing the risk of bronchopleural fistulas that typically require surgical intervention [35–37]. Besides detection it is also important to reliably evaluate treatment response in conservative therapy. Furthermore our data showed, that intravenous contrast seems not to be mandatory significantly for MRI and CXR at follow-up, indicating that CXR is comparable to MRI in regard of treatment response. However, in MRI the prevalence of abscess/necrosis decreased from 83.3% to 66.7%, whereas it did not change in CXR, implying that with decreasing size of abscess formation the assessment by CXR becomes increasingly difficult. Another reason could be the lower number of patients included in the follow-up group. These clinical considerations underline the relevance of a reliable detection of abscess formation/necrosis formation in children with complicated pneumonia for what MRI has the highest sensitivity.

The best intra- and interreader agreements were achieved for consolidation indicating a high diagnostic certainty in MRI and CXR. The prevalence of consolidation at baseline was not significantly different between MRI and CXR, whereas the severity of consolidation was rated higher in MRI. At follow-up the prevalence for consolidations did not change for MRI, whereas it decreased by -25% in CXR. The subscores for consolidation decreased significantly for MRI and CXR. In summary, MRI showed no significant advantages regarding the assessment of consolidation. Only in the setting of follow-up minor consolidations seem to slide below the detection limit of CXR. The clinical relevance of detecting such small lesions has to be critically discussed. Therefore in regard of consolidations there is no real benefit of MRI over CXR.

The scorings for pleural effusion/empyema were quite comparable to consolidation. At follow-up, the prevalence did not change for MRI, whereas it also decreased by -25% in CXR. This is due to the fact that a minimum of 150 ml is required to detect effusion in the erect position by CXR, whereas effusions as small as 5 ml can be detected with MRI or LUS. Nevertheless the clinical relevance of such small effusions is questionable. More clinical relevant is the distinction between empyema and parapneumonic effusion. Conservative management is usually sufficient in the case of abscess formation, while empyema needs to be treated with chest drain or by surgical debridement [8, 13]. The best time-point of surgical debridement is controversial, but early surgical interventions may reduce the duration of hospitalization [38]. The differentiation between both entities might be particularly difficult with CXR, especially regarding loculated effusions and peripheral lung abscesses [39]. In our study the prevalence of empyema was significantly higher in MRI, compared to CXR, with 74.1% and 11.1%, respectively. If additional LUS was performed the prevalence of empyema increased from 11.8% to 63.1%, which was nearly as good as for MRI. With respect to empyema, we feel that the administration of intravenous contrast is helpful to assess pleural inflammation. Nevertheless the typical septations within the pleural effusion, could be also very well demonstrated on the T2w Sequences even without the use of contrast media. As a consequence, contrast medium is not absolutely necessary for the diagnosis of pleural empyema, but may be required for the diagnosis of inflammatory pleural changes. In summary, MRI should be used if realization of LUS is not possible to exclude empyema.

The prevalence of bronchial wall thickening was higher in MRI than in CXR, with 97% and 27.3%, respectively. Furthermore, mucus plugging was only detected in MRI but not in CXR. This indicates, that MRI allows better evaluation of the accompanying airways disease, which seems to be limited by means of CXR. Both morphological findings had the lowest intra- and interreader agreements in MRI and CXR, suggesting that these findings are more prone to individual rating than consolidations and pleural effusions. Furthermore, Reader 2, who was less experienced in using the score, had a lower intrareader agreement than Reader 1. Therefore the reader's experience seems to have a particularly larger influence on the assessment of airways then on other morphological findings.

Our study has some limitations. The first one is the lack of a gold standard for comparing MRI and CXR. However, including CT, which is associated with more radiation exposure in pediatric patients, could not be ethically justified. Secondly, the comparison of a 3D imaging technique with a 2D might has some drawbacks, since the influence of summation effects on the scoring results should not be underestimated. For, example in MRI, pleural effusion/empyema was rated as score = 1 if effusion size was smaller than 1 cm and score = 2 if effusion size was larger than 1 cm. In CXR the scoring was depending on the percent size of the area affected on a single lung field: score = 0 (no abnormality), score = 1 (<50% of the lobe/lung-field involved), or score = 2 (≥50% of the lobe/lung-field involved). This might result in some kind of “underscoring” of pleural effusion in CXR, since a pleural effusion which is larger than 2cm not necessarily involves more than ≥50% of the lobe/lung. Finally, the time interval between the acquisition of the CXR and MRI was 1.45±1.42 days at baseline and 1.11±2.15 days at follow-up. This may reduce comparability of both imaging studies due to dynamics in the appearance of morphological findings, although 73% of the examinations were performed in a time interval of 1 day.

In conclusion, MRI demonstrates to be more sensitive than CXR in detecting abscess and empyema in complicated pneumonia, but it showed no advantages in detecting consolidation and pleural effusion. The combination of CXR and LUS can increase the sensitivity regarding abscesses and empyema but does not achieve completely the sensitivity level of MRI, especially regarding small and central abscesses without large accompanying consolidations. Furthermore our data showed, that intravenous contrast seems not to be mandatory, allowing to avoid potential side-effects due to the intravenous contrast application and keeping the imaging time short. MRI can contribute to a better clinical management of complicated pneumonia in hospitalized children and can potentially replace chest CT as a radiation-free modality. Some disadvantages of MRI in the clinical setting should be also addressed. The availability of MRI scanning time is still limited, and the need for sedation as well as the administration of contrast media with its possible side-effects has to be considered in non-cooperative young children.

## Supporting information

S1 Table MRI protocol. T1/T2 bSSFP = balanced T1/T2-weighted steady-state free precession (TrueFISP), T2 FSE = T2-weighted single-shot fast spin echo with half-Fourier acquisition (HASTE), T2 BLADE = T2-weighted periodically rotated overlapping parallel lines with enhanced reconstruction (BLADE), T1 GRE = T1-weighted gradient echo sequence (GRE), volume interpolated breath-hold acquisition (VIBE), Gd = i.v. injection of Gadolinum-based contrast material, tra = transversal plane, cor = coronary plane, sag = sagittal plane, fs = fat saturated, bh = breath-hold, TR = repetition time, TE = echo time, ST = slice thickness, FoV = Field of view.
(PDF)
