# Supplementary material for: The value of chest magnetic resonance imaging compared to chest radiographs with and without additional lung ultrasound in children with complicated pneumonia
Source: PLoS One. 2020 Mar 19;15(3):e0230252. doi: 10.1371/journal.pone.0230252 (PMC7082029; doi:10.1371/journal.pone.0230252)
Supplement: S1 Table — T1/T2 bSSFP = balanced T1/T2-weighted steady-state free precession (TrueFISP), T2 FSE = T2-weighted single-shot fast spin echo with half-Fourier acquisition (HASTE), T2 BLADE = T2-weighted periodically rotated overlapping parallel lines with enhanced reconstruction (BLADE), T1 GRE = T1-weighted gradient echo sequence (GRE), volume interpolated breath-hold acquisition (VIBE), Gd = i.v. injection of Gadolinum-based contrast material, tra = transversal plane, cor = coronary plane, sag = sagittal plane, fs = fat saturated, bh = breath-hold, TR = repetition time, TE = echo time, ST = slice thickness, FoV = Field of view. (PDF) [file pone.0230252.s001.pdf]

Table S1. MRI protocol.

Sequence	Specials	Plane	TR (ms)	TE (ms)	ST (mm)	Distance factor (%)	Slices	FoV (mm²)	Matrix	Voxel size (mm)	Scan time (min:s)
T1/T2 bSSFP	free breathing	cor	498.26	1.26	4.5	-60	120	400x400	256x256	1.6 x 1.6	2:08
T2 FSE	bh	tra	500.0	30	8.0	15	33	350x263	320x240	1.1 x 1.1	0:19
T2 FSE	bh	cor	500.0	28	8.0	0	30	400x400	320x320	1.3 x 1.3	0:17
T2 BLADE	multiple bh	tra	2760.0	113	6.0	30	43	420x420	256x256	1.6 x 1.6	2:03
T1 GRE fs	bh	tra	3.61	1.69	4.0	3D	88	400x300	320x240	1.3 x 1.3	0:19
T1 GRE	bh	tra	3.61	1.69	4.0	3D	88	400x300	320x240	1.3 x 1.3	0:18
T1 GRE	bh	cor	3.35	1.63	4.0	3D	56	400x400	288x288	1.4 x 1.4	0:16
T1 GRE fs	Bh, Gd	tra	3.61	1.69	4.0	3D	88	400x300	320x240	1.3 x 1.3	0:19
T1 GRE fs	Bh, Gd	cor	3.35	1.63	4.0	3D	56	400x400	288x288	1.4 x 1.4	0:17
